# Evaluation of reflux following sleeve gastrectomy and one anastomosis gastric bypass: 1-year results from a randomized open-label controlled trial

**DOI:** 10.1007/s00464-020-08182-3

**Published:** 2020-12-02

**Authors:** Mario Musella, Antonio Vitiello, Giovanna Berardi, Nunzio Velotti, Marcella Pesce, Giovanni Sarnelli

**Affiliations:** 1 Advanced Biomedical Sciences Department, Naples “Federico II” University, AOU “Federico II” - Via S. Pansini 5, 80131 Naples, Italy; 2 Clinical Medicine and Surgery Department, Naples “Federico II” University, AOU “Federico II” - Via S. Pansini 5, 80131 Naples, Italy

**Keywords:** Sleeve gastrectomy, Mini-bypass, One anastomosis gastric bypass, Gastroesophageal reflux, GERD

## Abstract

**Background:**

Recent reports have demonstrated that de novo reflux and worsening of pre-existing symptoms occur after SG; concerns are still expressed about the risk of symptomatic biliary reflux gastritis and oesophagitis. The aim of our study was to investigate and compare the rate of postoperative acid and non-acid reflux following Mini-/One anastomosis gastric bypass (MGB/OAGB) and laparoscopic sleeve gastrectomy (LSG).

**Study design:**

A prospective randomized open-label, controlled trial registered on clinicaltrial.gov (NCT number: NCT02987673) has been carried out to evaluate esophagogastric junction exposure to reflux in the first year after MGB/OAGB and LSG using high impedance manometry, endoscopy, and a validated questionnaire.

**Results:**

A total of 58 individuals were eventually enrolled in this trial and represented the per-protocol population (*n* = 28 MGB/OAGB, *n* = 30 LSG). No difference was found between the two groups in terms of demographic characteristics, PAGI-SYM score, acid exposure time percent of the esophagus (AET%), esophagitis, and other HRiM and MII-pH data at baseline. Comparing MII-pH outcomes of the two groups, AET% resulted significantly higher after LSG at 12 months. Endoscopic findings showed a significant increase of esophagitis ≥ B in the LSG group after 1 year; postoperative esophagitis ≥ B resulted also significantly worsened after LSG when compared to MGB/OAGB.

**Conclusion:**

Since AET% and rate of esophagitis are significantly higher after LSG when compared to MGB/OAGB, this procedure should be preferred in case of preoperative subclinical reflux or low grade (A) esophagitis.

Gastroesophageal reflux disease (GERD) prevalence is significantly higher in morbidly obese patients than in the general population [1]. The excess of visceral fat causes an increase of the intra-abdominal pressure, which reduces the efficacy of the lower esophageal sphincter (LES) and often induces the development of hiatal hernia (HH) [2]. Subsequently, massive weight loss is recommendable to improve heartburn and regurgitation in obese patients. Despite bariatric surgery is the most effective treatment for morbid obesity [3], controversial outcomes on GERD have been demonstrated due to the divergent effect of different procedures on LES and HH.

Sleeve Gastrectomy (SG) has rapidly become the most commonly performed bariatric surgery worldwide [4]. The fourth IFSO global registry report 2018 showed that SG accounted for 46% of all bariatric interventions, while the one anastomosis/mini gastric bypass (MGB/OAGB) reaches the 7.6% being the third procedure worldwide and the second in Eastern Europe, Asia, and the Middle East [5].

SG has been accepted with enthusiasm by the international community due to its laparoscopic feasibility, short learning curve, and satisfactory outcomes, which are comparable in the midterm to Roux-en-Y gastric bypass (RYGB) [6, 7]. On the other hand, even though MGB/OAGB provides shorter operative time and better results on weight loss and resolution of comorbidities than RYGB [8, 9], some criticism still resists around this procedure [10, 11]. Thousands of MGB/OAGBs have now been performed globally by several surgeons, but concerns are still expressed about the risk of symptomatic biliary reflux gastritis and oesophagitis [12]. Indeed, some authors believe Duodenal-Gastro-Esophageal Reflux (DGER) after MGB/OAGB increases the risk of Barrett’s esophagus (BE) and gastric/oesophageal cancer [13, 14].

Surprisingly, recent reports have demonstrated that de novo GERD and worsening of pre-existing symptoms occur after SG [15, 16]. Some studies have also described intestinal metaplasia (Barrett’s disease) after sleeve due to chronic acid exposure of the lower esophagus [17, 18].

High-resolution Impedance manometry (HRiM) and impedance-pH monitoring (MII-pH) are currently considered the gold standard to assess esophagogastric junction (GEJ) function, and exposure to reflux, especially after bariatric surgery [19, 20].

A prospective randomized open-label, controlled trial registered on clinicaltrial.gov (NCT number: NCT02987673) has been carried out to evaluate GEJ exposure to reflux in the first year after MGB/OAGB and LSG using HRiM and MII-pH, endoscopy and a validated questionnaire. Aim of our study was to investigate and compare the rate of postoperative acid and non-acid reflux following MGB/OAGB and LSG.

## Materials and methods

### Study design and inclusion criteria

In this prospective, open-label, superiority, randomized controlled trial, patients were recruited from January 2016 to August 2018 in our high-volume bariatric center at University hospital. The Institutional Review Board approved the study protocol (protocol no: 137/2016) and informed consent was obtained from each participant. The study was conducted according to the Helsinki declaration and was registered on clinicaltrial.gov (NCT number: NCT02987673). CONSORT checklist and apposite diagram were used to guide reporting of this study [21]

Inclusion criteria, according to international guidelines [22], were age between 18 and 65 years, body-mass index (BMI) ≥ 40 kg/m^2^, or ≥ 35 kg/m^2^ with the presence of at least one obesity-related disease (hypertension, type 2 diabetes, obstructive sleep apnoea, or dyslipidaemia).

Exclusion criteria from the study included clinical diagnosis of gastroesophageal reflux disease (GERD); previous abdominal surgery; paraesophageal (type 2), mixed (type 3), or sliding hiatal hernias ≥ 3 cm; presence of Barrett’s metaplasia and/or esophagitis grade ≥ B according to Lyon consensus [23]. In cases of intraoperative diagnosis of hiatal defects ≥ 3 cm, patients were excluded from the study.

### Randomization and masking

Patients were computer assigned one to one to MGB/OAGB or to the control group (LSG). Due to the necessity of informed consent, assignation was done the day of admission and the study was open-label without masking.

### Surgical technique

In order to minimize the bias related to the different surgical techniques, procedures performed according to standardized criteria [24] and all interventions were realized by the same surgeon (MM).

MGB/OAGB was routinely performed with a six-port laparoscopic technique. Patient was placed in the reverse Trendelenburg position. At the beginning of the operation, the surgeon stands between the patient's legs in order to prepare the phrenogastric ligament then, in some cases, moves to the right side of the patient. The gastric pouch was constructed by applying one horizontal 45-mm linear stapler at the angle of the lesser curvature, just below the left branch of the crow’s foot. The pouch was calibrated along a 36-Fr tube. No reinforcement was routinely applied on the staple line. The bypassed jejunum was measured with a graded grasper at a length ranging from 180 from 220 cms from ligament of Treitz depending on the preoperative BMI of patients [25]. Those with preoperative BMI < 45 had a biliopancreatic limb (BLL) of 180 cm, while for those with a BMI between 45 and 50 the BLL was 200 cm; in the case of BMI > 50 BLL was 220 cm. The gastrojejunostomy was performed using a 45-mm linear stapler and enterotomies were closed by an anterior, double-layer, self-locking, running absorbable suture (V-lock 3/0, Medtronic™, Minneapolis, U.S.A.). All the anastomoses were performed at least 13 cm distally to the GEJ (14.9 ± 1.1).

LSG begins with the vascular preparation of the greater gastric curvature starting 5–6 cm proximal to the pylorus and going up to the His angle. A 36-Fr bougie was placed transorally down to the pyloric channel, then a vertical gastrectomy was performed with a linear stapler. In both surgeries, suture lines and anastomoses were tested intraoperatively by methylene-blue injection through the bougie.

### Outcomes and follow-up

Primary endpoint was lower esophagus acid exposure as percentage of time (AET%) at 6 and 12 months evaluated with high-resolution impedance manometry (HRiM) and impedance-pH monitoring (MII-pH); secondary endpoints at 6 and 12 months were Percentage of Excess BMI Loss (EBMIL%), defined as (final BMI–initial BMI)/(initial BMI–25), scores of Patient Assessment of Gastrointestinal Disorders–Symptom Severity Index (PAGI-SYM) standardized questionnaire and rate of esophagitis.

### HRiM and MII-pH

Each studied patient underwent a full manometric assessment according to standardized protocol, including ten 5-ml wet swallows and multiple rapid swallows. Briefly, a 36-channel solid-state manometric assembly with 1-cm spacing pressure sensors was used (Manoscan; Sierra Scientific Instruments Inc, Los Angeles, CA). The recorded pressure data were evaluated after thermal compensation with Manoview software (Sierra Scientific Instruments Inc, Los Angeles, CA) prior to data analysis. The HRM assembly was placed transnasally and the manometric catheter positioned to record from the hypopharynx to the stomach. All studies were performed in a supine position after at least a 6-h fasting and esophageal motility was classified according to standardized criteria, using Chicago classification [26].

Following manometric assessment, all patients underwent an OFF therapy 24-h ambulatory multichannel intraluminal impedance-pH monitoring (24 h pH-MII). The pH probe was placed under manometric guidance in order to properly assess the position of the lower esophageal sphincter (LES). The system included a portable data logger and a catheter with six impedance electrodes at 3, 5, 7, 9, 15, and 17 cm above the LES and two antimony pH electrodes located at 5 cm above and 10 cm below the LES, respectively (Sleuth Multi-Channel Intraluminal Impedance ambulatory system; Sandhill Scientific, Inc.; Highland Ranch, CO). Data were stored on a compact-flash card and were analyzed using a specific diagnostic software (BioVIEW, Sandhill Scientific). In all patients, the number, proximal propagation and characterization of reflux events (acid reflux: pH < 4, weakly acid reflux: 4 < pH < 7, and weakly alkaline reflux: pH > 7), total esophageal acid exposure, intragastric pH and percentage of time that gastric pH was less than 4 were recorded. Six and twelve months after surgery all patients underwent a re-assessment of manometrical and pH-impedance parameters by using the same protocol.

### Upper endoscopy

Endoscopy was performed off therapy after an 8-h fast using an endoscope (Olympus CV 150-Tokyo, Japan). Esophagitis was described following Los Angeles classification [27]. Endoscopy was performed preoperatively and after 1 year by the same endoscopist, blinded for patient symptoms.

### PAGI-SYM standardized questionnaire

The PAGI-SYM contains 20 items with six subscales: (1) nausea/vomiting; (2) fullness/early satiety; (3) bloating; (4) upper abdominal pain; (5) lower abdominal pain; and (6) heartburn/regurgitation. For the PAGI-SYM, items are scored on a 6-point Likert scale, with response options ranging from 0 (none) to 5 (very severe), and subscale scores calculated by averaging relevant item responses within the subscale as specified in Rentz et al. [28]. Total scores are also calculated by averaging the subscale scores. Negative changes (from baseline) indicate improvement or a decrease in severity of symptoms on the PAGI-SYM. The same evaluation was repeated in a follow-up (FU) period of both 6 months and 12 months after surgery.

### Statistical analysis

Sample size was calculated based on previous evidences [20] of HRiM and MII-pH in MGB/OAGB and LSG and using the following formula:

$$ n = \, 2\left( {Z_{2\alpha } + \, Z_{\beta } } \right) \times {{\sigma^{2} } \mathord{\left/ {\vphantom {{\sigma^{2} } {\Delta^{2} }}} \right. \kern-\nulldelimiterspace} {\Delta^{2} }} \, $$

In order to observe a clinically significant difference (Δ) $\ge$ 3%, assuming a standard deviation (σ) of 4% with a statistical power of 80%, an α risk of 5%, 28 patients per arm (56 in total) were required. Due to the invasiveness and discomfort of HRiM and MII-pH, we had initially planned 40 participants per group, but, considering a drop-out up to 20%, at least 32 subjects per group were necessary. Primary and secondary efficacy outcomes were analyzed in the per-protocol population, which included all patients randomly assigned to surgery who contributed data and excluded those with major deviations from the protocol (withdrawal of consent, intraoperative diagnosis of HH). Comparisons were made using Student’s t test with a bilateral 95% confidence interval for the mean difference (two-sided 5% α level) and Pearson’s chi-square or Fisher’s exact test for categorical variables. The analysis was performed with SPSS version 20.0 (IBM, Armonk, NY).

## Results

A total of 64 obese patients, 32 in each group, were initially included in the study. Four subjects from MGB/OAGB group were excluded: 1 patient was intraoperatively diagnosed with hiatal hernia while 3 subjects refused to undergo postoperative evaluation. Two patients from LSG group missed 12 months of assessment and were subsequently excluded. A total of 58 (93.5%) individuals were eventually enrolled in this trial and represented the per-protocol population (*n* = 28 MGB/OAGB, *n* = 30 LSG). No difference was found between the two groups in terms of demographic characteristics, PAGI-SYM score, AET%, esophagitis, and other HRiM and MII-pH data at baseline (Table1).

**Table 1:** Comparison of HRiM and MII-pH data between the two groups at baseline

	LSG	MGB/OAGB	*p* value
BMI (KG/m^2^)	47.5 ± 7.3	48.5 ± 8.8	ns
PAGI-SYM score	0.6 ± 1	0.7 ± 1.4	ns
Acid exposure time (%)	4.3 ± 9.4	3.3 ± 4	ns
Distal reflux (*n*.)	63.5 ± 33.6	52.4 ± 25.7	ns
Distal acid reflux (*n*.)	40.3 ± 35.7	46.8 ± 21.1	ns
Non-acid distal reflux (*n*.)	27 ± 23.4	22.3 ± 25.6	ns
Proximal reflux (*n*.)	32.6 ± 17.4	30.6 ± 21.3	ns
Acid proximal reflux (*n*.)	17.5 ± 14	17.3 ± 14.1	ns
Non-acid proximal reflux	14.4 ± 14	12.5 ± 18.5	ns
LES tone (mmHg)	17.4 ± 4.7	18 ± 5.7	ns

Data are expressed as mean ± standard deviation

*ns* not significant

In the MGB/OAGB group, we found a progressive decrease of AET% and a significant reduction of distal acid reflux episodes both at 6 months and 12 months after surgery; there was also a significant reduction of LES tone at 6 months but not after 1 year (Table 2). No significant increase of non-acid refluxes was demonstrated at any time of follow-up.

**Table 2:** Comparison of HRiM and MII-pH data in MGB/OAGB group between baseline and follow-up

	Baseline MGB/OAGB	6 months FU MGB/OAGB	6 months *p* value	12 months FU MGB/OAGB	12 months *p* value
Acid exposure time (%)	3.3 ± 4	1.7 ± 2.3	ns	1.5 ± 2.3	ns
Distal reflux (*n*.)	52.4 ± 25.7	54,9 ± 38.9	ns	54.5 ± 20.7	ns
Distal acid reflux (*n*.)	46.8 ± 21.1	21,8 ± 18.9	< 0.01	23 ± 21.2	< 0.01
Non-acid distal reflux (*n*.)	22.3 ± 25.6	33.2 ± 26.2	ns	31 ± 17.4	ns
Proximal reflux (*n*.)	30.6 ± 21.3	29.2 ± 22	ns	30.4 ± 14	ns
Acid proximal reflux (*n*.)	17.3 ± 14.1	12.3 ± 9.9	ns	12.9 ± 13.6	ns
Non-acid proximal reflux	12.5 ± 18.5	16.7 ± 16	ns	16.4 ± 9.5	ns
LES tone (mmHg)	18 ± 5.7	13.3 ± 6.1	< 0.05	15.6 ± 8.3	ns

Data are expressed as mean ± standard deviation

*ns* not significant

In the LSG group, we found no significant increase or decrease of MII-pH data after 6 and 12 months (Table 3). However, a progressive increase of AET% in the postoperative period was recorded.

**Table 3:** Comparison of HRiM and MII-pH data in LSG group between baseline and follow-up

	Baseline LSG	6 months FU LSG	6 months *p* value	12 months FU LSG	12 months *p* value
Acid exposure time (%)	4.3 ± 9.4	4.7 ± 7.6	ns	7.1 ± 6.8	ns
Distal reflux (*n*.)	63.5 ± 33.6	74.6 ± 57	ns	62.7 ± 38.4	ns
Distal acid reflux (*n*.)	40.3 ± 35.7	36.1 ± 34.9	ns	40.1 ± 30	ns
Non-acid distal reflux (*n*.)	27 ± 23.4	42.1 ± 38	ns	22.3 ± 13.7	ns
Proximal reflux (*n*.)	32.6 ± 17.4	40.3 ± 29.5	ns	35.2 ± 20.6	ns
Acid proximal reflux (*n*.)	17.5 ± 14	27.2 ± 38.3	ns	19.6 ± 14.3	ns
Non-acid proximal reflux	14.4 ± 14	20.7 ± 20.3	ns	14.4 ± 9.5	ns
LES tone (mmHg)	17.4 ± 4.7	18 ± 5.2	ns	21.2 ± 11.2	ns

Data are expressed as mean ± standard deviation

*ns* not significant

**Fig. 1:**
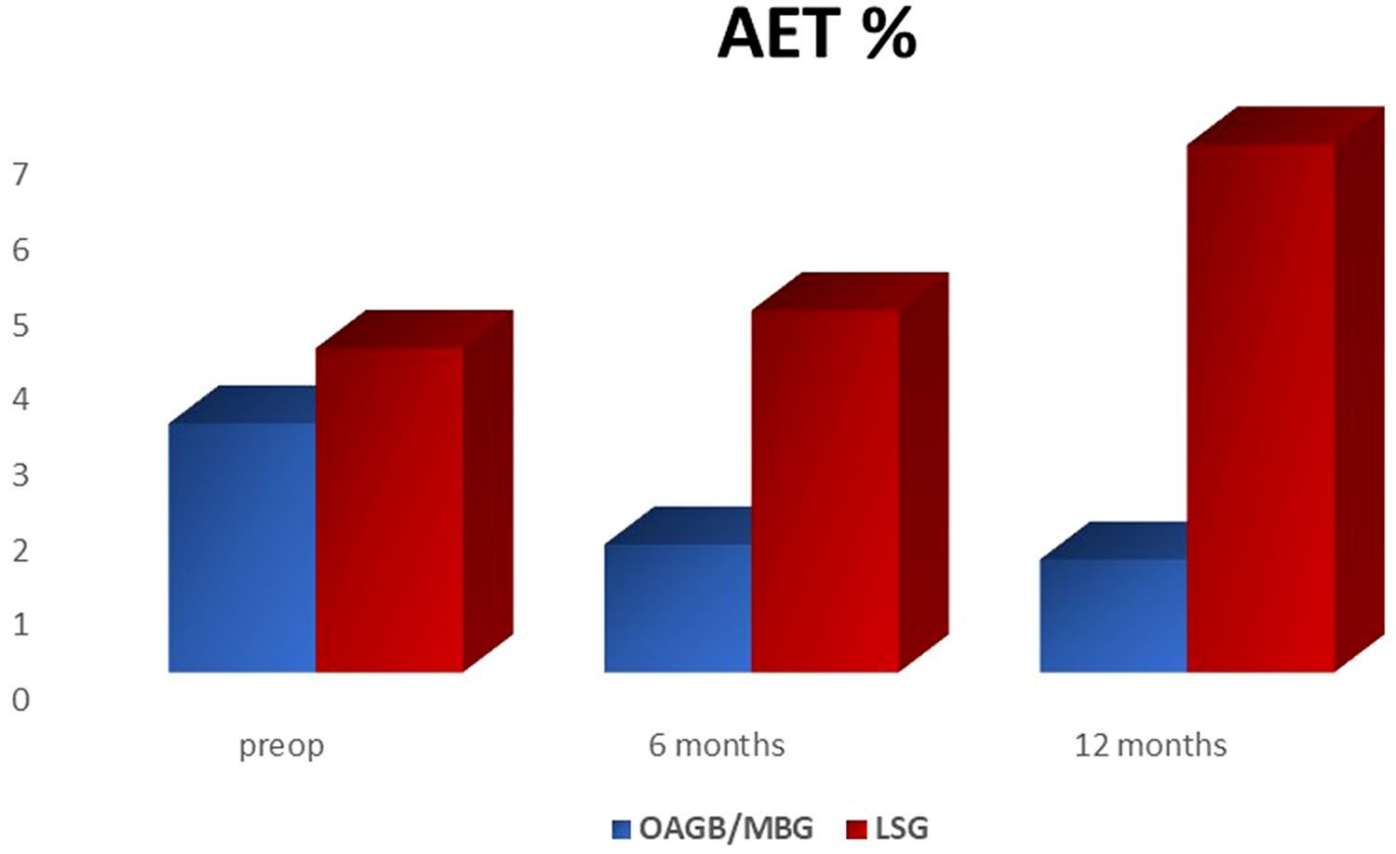
Comparison of esophagus acid exposure as percentage of time (AET%) during follow-up. *P* < 0.01 at 12 months

Comparing MII-pH outcomes of the two groups, AET% resulted significantly higher after LSG at 12 months, while LES tone was significantly higher at 6 months but not at 12 months (Table 4, Fig. 1).

**Table 4:** Comparison of HRiM and MII-pH data between MGB/OAGB and LSG group at follow-up

	6 months FU MGB/OAGB	6 months FU LSG	*p* value	12 months FU MGB/OAGB	12 months FU LSG	*p* value
Acid exposure time (%)	1.7 ± 2.3	4.7 ± 7.6	ns	1.5 ± 2.3	7.1 ± 6.8	< 0.01
Distal reflux (*n*.)	54.9 ± 38.9	74.6 ± 57	ns	54.5 ± 20.7	62.7 ± 38.4	ns
Distal acid reflux (*n*.)	21.8 ± 18.9	36.1 ± 34.9	ns	23 ± 21.2	40.1 ± 30	ns
Non-acid distal reflux (*n*.)	33.2 ± 26.2	42.1 ± 38	ns	31 ± 17.4	22.3 ± 13.7	ns
Proximal reflux (*n*.)	29.2 ± 22	40.3 ± 29.5	ns	30.4 ± 14	35.2 ± 20.6	ns
Acid proximal reflux (*n*.)	12.3 ± 9.9	27.2 ± 38.3	ns	12.9 ± 13.6	19.6 ± 14.3	ns
Non-acid proximal reflux	16.7 ± 16	20.7 ± 20.3	ns	16.4 ± 9.5	14.4 ± 9.5	ns
LES tone (mmHg)	13.3 ± 6.1	18 ± 5.2	< 0.01	15.6 ± 8.3	21.2 ± 11.2	ns

Data are expressed as mean ± standard deviation

*ns* not significant

PAGI-SYM questionnaire revealed no significant worsening of symptoms at 6 and 12 months both after MGB/OAGB and LSG; mean postoperative scores were also comparable between patients of the two groups (Table 5).

**Table 5:** PAGI-SYM score

	initial score	6 months	*p* value	12 months	*p* value
LSG	0.56 ± 1.04	0.38 ± 0.90	ns	0.55 ± 1.22	ns
MGB/OAGB	0.67 ± 1.41	0.31 ± 1.02	ns	0.13 ± 0.44	ns
*p* value	ns	ns		ns	

Data are expressed as mean ± standard deviation

*ns* not significant

Weight loss was comparable between the two procedure at any time of follow-up (Table 6).

**Table 6:** Weight loss expressed as Body Mass Index (BMI) and percentage of excess Body Mass Index Loss (EBMIL%)

	LSG	MGB/OAGB	*p* value
Initial BMI	47.5 ± 7.3	48.5 ± 8.9	ns
BMI at 6 months (kg/m^2^)	36.1 ± 5.5	35.9 ± 5.3	ns
BMI at 12 months (kg/m^2^)	30 ± 4.3	31.3 ± 4.6	ns
EBMIL% at 6 months	49.6 ± 24.1	50.6 ± 16	ns
EBMIL% at 12 months	77.6 ± 18.8	80.3 ± 33.9	ns

Data are expressed as mean ± standard deviation

**Fig. 2:**
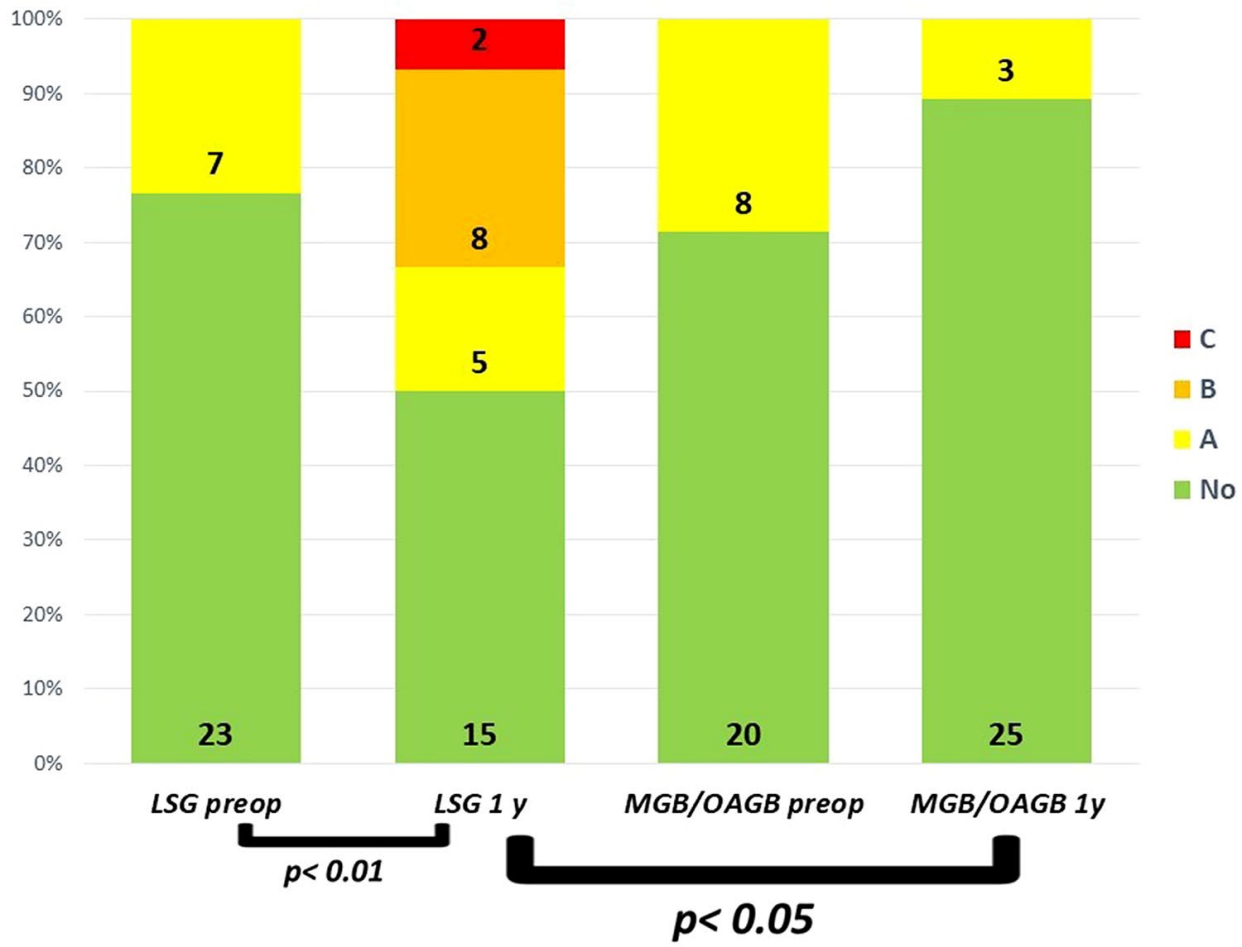
Prevalence of reflux esophagitis ≥ B before and 1 year after LSG and MGB/OAGB

Endoscopic findings showed a significant increase of esophagitis ≥ B in the LSG group after 1 year; postoperative esophagitis ≥ B resulted also significantly worsened after LSG when compared to MGB/OAGB (Fig. 2).

## Discussion

Theoretically, SG should improve GERD symptoms. Indeed, the surgical procedure provides removal of the body and fundus, which are the acid-producing part of the stomach. However, with the exception of few studies [29, 30], the vast majority of published evidence demonstrates a worsening of pre-existing GERD and new onset disease [31, 32] after SG. A recent systematic review showed 20% of de novo GERD [33], while a meta-analysis found that the increase of postoperative GERD after sleeve was 19% and de novo reflux was 23% [34]. Some authors also showed a significant increase in the use of antacid medication after this procedure [35, 36]. Several reports of BE due to chronic reflux after SG have also been published. Even if Braghetto [37] first described an incidence of 1.2% of BE already 1 year after SG, other authors reported a rate of 15–17% after a longer follow-up [17, 18]. Soricelli et al. [38] showed that BE after SG arise in asymptomatic patients in 21% of cases. On the contrary, in a recent multicenter study [39] only one patient with Barrett’s metaplasia was not complaining of GERD symptoms; authors also stressed that they found only short BE and no case of dysplasia was recorded. Indeed, in non-dysplastic BE, risk of progression to cancer, according to Desai et al., is 0.33% annually [40], while malignant transformation annual rate in low- and high-grade dysplastic BE is 0.5% and 7%, respectively [41, 42]. Several mechanisms could be responsible for reflux after SG. First, a sleeved stomach has an increased intragastric pressure (IGP) and decreased compliance. Second, extensive dissection of the phrenoesophageal ligament or incomplete resection of the fundus may cause reflux through reduced LES pressure and persistence of acid production. Moreover, defective surgical technique could lead to a twisted or stenotic sleeve with subsequent obstruction of the stomach and regurgitation of acid content into the esophagus. Undiagnosed HH or intrathoracic migration of the gastric tube have been also accounted as other possible causes of postoperative GERD [43, 44]. However, recent studies have also demonstrated that HH repair could not improve or prevent symptoms [45, 46]. Some authors have also reported delayed gastric emptying after LSG, which could be another cause of reflux of gastric content into the stomach [47–50].

Due to its omega loop reconstruction, many authors have claimed that MGB/OAGB, in the long term, could increase the risk of bile reflux and cancer [11, 49]. However, since first publication from Rutledge [50], symptomatic biliary gastritis and oesophagitis, requiring revisional surgery, have been reported rarely in the literature [51, 52]. No report of BE after MGB/OAGB is currently available, while sporadic cases of cancer have been published [53, 54]. However, no preoperative endoscopy was available and surgical technique may have been defective in these cases [55]. Criticism around MGB/OAGB comes from loop fashion and its presumed resemblance to Billroth II operation; previous publications have enlightened that MGB/OAGB, when correctly performed, has a long narrow gastric pouch with the gastro-jejunal anastomosis placed at least 13 cm distally from GEJ [56]. These differences are crucial to reduce the risk of alkaline reflux into the esophagus, as demonstrated using HRiM and MII-pH.

Several studies with HRiM and MII-pH have also shown different effects of SG and MGB/OAGB on GEJ and IGP [57, 58]. These reports demonstrated that after SG the narrow stomach behaves as a poorly dilatable cylinder between the LES and the pylorus; subsequently the high IGP increases the risk of reflux into the esophagus. Conversely, after MGB/OAGB, even if the IGP is increased in the sleeve-shaped pouch, the presence of a distal anastomosis allows the bile to flow down into jejunum.

Our outcomes show interesting results when compared to previously published studies. In the LSG arm, there was no significant increase of AET% or other HRiM and MII-pH evidences of acid reflux in the first postoperative year. Moreover, clinical evaluation with PAGI-SYM did not demonstrate worsening of symptoms during the follow-up period. This finding was probably due to massive weight loss and subsequent reduction of intra-abdominal pressure, which reaches the nadir in the first postoperative twelve months. However, a significant increase of esophagitis was already diagnosed after 12 months.

Conversely, in the MGB/OAGB group, two paramount results come from our study. First, even if not significantly, AET% reduces after the procedure while number of episodes of distal acid reflux were both significantly reduced after 6 and 12 months; second, no significant increase of non-acid reflux was recorded. PAGI-SYM questionnaire and endoscopy showed no worsening of symptoms and esophagitis during the follow-up period.

Our data show clearly that MGB/OAGB has a better effect on all kind of reflux when compared to LSG. These outcomes could demonstrate that the Roux reconstruction is not the only technique able to reduce acid and non-acid reflux into the esophagus, as commonly believed.

The absence of significant difference in weight loss between the two procedures was not unexpected, since LSG shows satisfactory results in the first twelve months and weight regain occurs later in the FU [59]. Moreover, greater weight loss for one of the procedures could have been a potential bias, whereas it could have led to significant reduction of intra-abdominal pressure and subsequent effect on LES function. Specifically, our data could demonstrate that GERD improvement after MGB/OAGB is independent from weight loss and it may be caused by the abovementioned surgical reconstruction, which facilitates the passage of gastric content into the jejunum. On the other hand, after LSG, the increased IGP is initially balanced by massive weight loss but worsening of GERD and risk of BE may increase during the follow-up due to weight regain. Our study also enlightens a paradoxical relationship between LES pressure and reflux after LSG and MGB/OAGB. As said before, a defective technique may cause division of the sling fibers provoking a decrease in the LES resting pressure. However, De novo GERD may arise also in patients with a properly performed procedure and high LES pressure due to reduced gastric emptying [47, 48]. This could explain why we found higher LES pressure but increased AET% at 1 year after LSG. On the contrary, MGB/OAGB reduces LES pressure due to the presence of biliary acid [60, 61] into the stomach without causing alkaline reflux.

In the light of what has been reported by previous authors [17, 18], who have demonstrated an increased rate of BE at 5 years after LSG, our findings could suggest that the mechanism of BE development starts already at 1 year after LSG. Increased acid exposure of the esophagus may lead to initial mucosal injury in the first 12 months, while subsequent overlap of alkaline reflux could be the reason of degeneration to BE in the longer period [62].

## Strengths and limitations

To the best of our knowledge, this is the first study reporting results of a prospective randomized controlled trial comparing specifically GEJ function after MGB/OAGB and SG with 1-year FU [63]. Main strength of this study is a full evaluation with endoscopy, manometry, and clinical assessment of GERD. Indeed, HRiM and MII-pH and endoscopy are considered the gold standard for the assessment of esophageal function and reflux [19, 22]. Main limitation is that data are collected from a single center. Moreover, even if a comparison between RYGB and MGB/OAGB would have been more interesting, when we started the trial GERD after LSG was a less-known postoperative problem.

## Conclusion

Prospective evaluation of reflux with HRiM and MII-pH and a validated clinical questionnaire demonstrated that, in the first postoperative year, acid reflux does not significantly worsen both after MGB/OAGB and LSG. However, rate of esophagitis ≥ B increased 12 months after LSG. Non-acid reflux does not increase after MGB/OAGB, as commonly believed. Since acid exposure time percent of the esophagus (AET%) and rate of esophagitis are significantly higher after LSG when compared to MGB/OAGB, this procedure should be preferred in the case of preoperative GERD or low-grade (A) esophagitis.
